# Evaluation of the Efficacy of a Smoking Cessation Intervention for Cervical Cancer Survivors and Women With High-Grade Cervical Dysplasia: Protocol for a Randomized Controlled Trial

**DOI:** 10.2196/34502

**Published:** 2021-12-30

**Authors:** Sarah R Jones, Damon J Vidrine, David W Wetter, Ya-Chen Tina Shih, Steven K Sutton, Lois M Ramondetta, Linda S Elting, Joan L Walker, Katie M Smith, Summer G Frank-Pearce, Yisheng Li, Vani N Simmons, Jennifer I Vidrine

**Affiliations:** 1 Tobacco Research & Intervention Program Department of Health Outcomes and Behavior Moffitt Cancer Center Tampa, FL United States; 2 Huntsman Cancer Institute Department of Population Health Sciences University of Utah Salt Lake City, UT United States; 3 Department of Health Services Research The University of Texas MD Anderson Cancer Center Houston, TX United States; 4 Department of Biostatistics and Bioinformatics Moffitt Cancer Center Tampa, FL United States; 5 Department of Gynecologic Oncology and Reproductive Medicine The University of Texas MD Anderson Cancer Center Houston, TX United States; 6 Department of Obstetrics and Gynecology University of Oklahoma Health Sciences Center Oklahoma City, OK United States; 7 Stephenson Cancer Center University of Oklahoma Health Sciences Center Oklahoma City, OK United States; 8 Department of Biostatistics and Epidemiology College of Public Health University of Oklahoma Health Sciences Center Oklahoma City, OK United States; 9 TSET Health Promotion Research Center Stephenson Cancer Center University of Oklahoma Health Sciences Center Oklahoma City, OK United States; 10 Department of Biostatistics The University of Texas MD Anderson Cancer Center Houston, TX United States

**Keywords:** smoking cessation, cervical cancer, cancer survivor, motivation, tobacco treatment, cancer, smoking, RCT, randomized controlled trial, cognitive behavior, intervention

## Abstract

**Background:**

The prevalence of smoking among cervical cancer survivors is strikingly high, yet no smoking cessation interventions to date have specifically targeted this population. This paper describes the study design, methods, and data analysis plans for a randomized clinical trial designed to evaluate the efficacy of a theoretically and empirically based Motivation And Problem Solving (MAPS) approach for promoting and facilitating smoking cessation among cervical cancer survivors. MAPS is a comprehensive, dynamic, and holistic intervention that incorporates empirically supported cognitive behavioral and social cognitive theory–based treatment strategies within an overarching motivational framework. MAPS is designed to be appropriate for all smokers regardless of their motivation to change and views motivation as dynamically fluctuating from moment to moment throughout the behavior change process.

**Objective:**

This 2-group randomized controlled trial compares the efficacy of standard treatment to MAPS in facilitating smoking cessation among women with a history of high-grade cervical dysplasia or cervical cancer.

**Methods:**

Participants (N=202) are current smokers with a history of high-grade cervical dysplasia or cervical cancer recruited nationally and randomly assigned to one of two treatment conditions: (1) standard treatment (ST) or (2) MAPS. ST consists of repeated letters referring participants to their state’s tobacco cessation quitline, standard self-help materials, and free nicotine replacement therapy when ready to quit. MAPS has all ST components along with 6 proactive telephone counseling sessions delivered over 12 months. The primary outcome is abstinence from tobacco at 18 months. Secondary outcomes include abstinence over time across all assessment points, abstinence at other individual assessment time points, quit attempts, cigarettes per day, and use of state quitlines. Hypothesized treatment mechanisms and cost-effectiveness will also be evaluated.

**Results:**

This study was approved by the institutional review boards at the University of Texas MD Anderson Cancer Center, the University of Oklahoma Health Sciences Center, and Moffitt Cancer Center. Participant enrollment concluded at Moffitt Cancer Center in January 2020, and follow-up data collection was completed in July 2021. Data analysis is ongoing.

**Conclusions:**

This study will yield crucial information regarding the efficacy and cost-effectiveness of a MAPS approach for smoking cessation tailored to the specific needs of women with a history of high-grade cervical dysplasia or cervical cancer. Findings indicating that MAPS has substantially greater efficacy than existing evidence-based tobacco cessation treatments would have tremendous public health significance.

**Trial Registration:**

ClinicalTrials.gov NCT02157610; https://clinicaltrials.gov/ct2/show/NCT02157610

**International Registered Report Identifier (IRRID):**

DERR1-10.2196/34502

## Introduction

The incidence of cervical cancer in the United States declined by more than half between 1975 (14.8 per 100,000 population) and 2018 (6.7 per 100,000 population) [1] owing to the widespread uptake of screening, primarily with the Pap test. Owing to early detection, mortality has also declined substantially. Despite these declines, 14,480 new cases of cervical cancer are expected to be diagnosed and 4290 women are estimated to die from the disease in 2021 [2]. As of January 2019, there were estimated to be approximately 288,710 cervical cancer survivors in the United States [3,4]. Furthermore, there are profound racial/ethnic and sociodemographic disparities in the incidence and mortality of cervical cancer [4-9].

Smoking is a well-established risk factor for cervical intraepithelial neoplasia (CIN), also known as high-grade cervical dysplasia [10], which is the immediate precursor to cervical cancer. Women with a history of CIN have a considerably higher risk of developing cervical cancer [11]. Furthermore, nationwide data indicate that cervical cancer survivors have among the highest rates of continued smoking post diagnosis—between 30% and 48% [12-14]. Continued smoking is associated with several adverse outcomes including increased cancer recurrence, increased risk of a secondary malignancy, poor treatment outcomes, and decreased quality of life [15-20]. Hence, it has been recommended that cervical cancer survivors receive a survivorship care plan that addresses the dangers of continued tobacco use and the risk of subsequent malignancy that persists throughout the survivor’s lifetime. A crucial part of this survivorship plan should involve the delivery of smoking cessation treatment designed to address the specific needs of these women [21].

No known smoking cessation interventions have specifically targeted cervical cancer survivors. This study is, to our knowledge, the first to evaluate the efficacy and cost-effectiveness of an intervention designed to address the specific needs of this population. Motivation And Problem Solving (MAPS) is a holistic, dynamic approach to facilitating and maintaining behavior change that utilizes a combined motivational enhancement and social cognitive approach based on motivational interviewing (MI) [22,23] and social cognitive theory [24,25]. Because the MAPS approach is built around a wellness program that addresses numerous barriers and concerns prevalent among cervical cancer survivors (eg, anxiety, depression, stress, and fear of cancer recurrence), we believe it is appropriate for treating this population. In addition, previous research has supported the efficacy of MAPS in diverse populations of smokers for motivating quit attempts, increasing cessation, and preventing relapse [26-28].

This paper describes the research design, methods, and data analysis plans for an ongoing randomized controlled trial (RCT) designed to evaluate the efficacy of MAPS in facilitating smoking cessation among high-grade cervical dysplasia and cervical cancer survivors. The primary aim is to compare the efficacy of MAPS in facilitating smoking cessation with standard treatment (ST) among women with a history of high-grade cervical dysplasia or cervical cancer. Secondary aims include (1) evaluating the effects of MAPS on hypothesized treatment mechanisms (motivation, agency, and stress/negative affect) and the role of those mechanisms in mediating MAPS effects on abstinence, and (2) assessing the cost-effectiveness of MAPS compared with ST (Multimedia Appendix 1).

## Methods

### Study Design

This RCT comprises 2 treatment arms. The ST group receives a mailed packet with a letter referring participants to their state’s tobacco cessation quitline, standard self-help materials, and free nicotine replacement therapy (NRT) when ready to quit. These treatment components are delivered at three timepoints: baseline, 6 months, and 12 months. MAPS has all ST components plus 6 proactive telephone counseling sessions delivered over 12 months. The timing of the telephone counseling sessions is flexible and determined jointly by the participant and the counselor. Assessments are conducted via telephone at baseline, 3, 6, 12, and 18 months. The primary outcome is 7-day point prevalence abstinence from tobacco at 18 months. Secondary outcomes are abstinence from tobacco at all other assessment points (3, 6, and 12 months), quit attempts, cigarettes per day, use of the state quitline, and cost-effectiveness.

We hypothesized that MAPS participants will have higher rates of smoking abstinence at 18 months than ST participants. Similarly, for secondary outcomes, we hypothesized that MAPS participants will have higher rates of smoking abstinence across all assessment points, more quit attempts, fewer cigarettes per day (when smoking), and greater use of the state quitline. Secondary hypotheses were that MAPS will (1) lead to higher abstinence rates through influencing the treatment mechanisms of motivation, agency, and stress/negative affect; and (2) be more cost-effective.

### Participants

Participants (target sample size, n=300) are women with a history of high-grade cervical dysplasia or cervical cancer, recruited via the following: (1) a gynecologic oncology clinic within a National Cancer Institute–designated cancer center in the South Central United States, (2) a university-based women’s health clinic, (3) a university-based tobacco treatment program, and (4) nationally via Facebook and paid Google search advertisements. Inclusion criteria are (1) being aged 18 years or older; (2) self-reporting smoking within the last 30 days and having a history of at least 100 lifetime cigarettes; (3) having a history of high-grade cervical dysplasia or cervical cancer; (4) having a working cell phone; (5) having a valid home address; and (6) being able to speak English, Spanish, or both languages. Exclusion criteria are (1) current use of NRT or other smoking cessation medications (eg, varenicline or bupropion), (2) being pregnant or breastfeeding, (3) having another household member enrolled in the study, or (4) having a contraindication of nicotine patch use.

### Procedures

This study was reviewed and approved by the University of Texas MD Anderson Cancer Center institutional review board (IRB), the University of Oklahoma Health Sciences Center IRB, and Advarra (H. Lee Moffitt Cancer Center IRB), and is registered on ClinicalTrials.gov (NCT02157610).

Potentially eligible women recruited in clinic were identified through electronic health record reviews and approached by research staff in person during medical visits or contacted via telephone. Participants referred by the tobacco treatment program were screened by research staff via telephone. Participants recruited via Facebook and Google paid search ads were initially directed to a Research Electronic Data Capture (REDCap) screener and asked to complete a brief set of screening questions. REDCap is a secure, web-based application designed to support data capture and utilizes a computer-administered self-interview format. This system is designed to comply with all Health Insurance Portability and Accountability Act (HIPAA) regulations. Those who passed the initial screening criteria were asked to provide their contact information so that they could be contacted by a research coordinator for further screening. All eligible women were invited to participate. A detailed description of the study was provided, and those who agreed to enroll completed an informed consent process either in person or over the telephone. Women who declined or were ineligible were offered self-help materials and a referral to other cessation programs.

Individuals who meet eligibility criteria and provide informed consent complete the baseline assessment over the telephone with a research coordinator or via a secure electronic REDCap link sent via email or text message. Participants are then randomized to ST or MAPS using a form of adaptive randomization called minimization [29,30]. Compared with techniques such as stratification, minimization results in better group balance with respect to participant characteristics. Minimization also provides balanced treatment groups throughout the randomization process. Thus, the treatment groups remain balanced with respect to participant characteristics that may be related to time of accrual. Variables for the minimization were race/ethnicity (nonminority or minority), age (≤35 or >35 years), education (<high school/general education development or ≥ high school/general education development), cigarettes per day (≤19 or ≥20), diagnosis at study enrollment (high-grade cervical dysplasia, stage 1 or 2, stage 3, or stage 4), treatment status (in active treatment or completed treatment), and time since diagnosis (≤1 year or >1 year). Following randomization, participants are mailed the appropriate intervention materials. Twelve weeks of combination NRT (patch + lozenge) are sent via mail when ready to quit.

Follow-up assessments occur at 3, 6, 12, and 18 months. Participants are provided the option to complete the assessment over the telephone with a research coordinator or on the internet via a secure REDCap link. Participants receive US $30 of compensation for completing the baseline assessment and US $30 for each completed follow-up assessment. In addition, participants receive US $30 at the baseline and all follow-up assessments to compensate for use of their personal cell phones for study participation. Participants may also be compensated US $30 at the 3-, 6-, 12-, and 18-month assessments for returning saliva cotinine tests to biochemically confirm smoking status.

### Intervention Conditions

#### Standard Treatment

ST consists of a mailed packet of materials including a letter referring smokers to their state’s tobacco cessation quitline, NRT when ready to quit, and standard self-help materials (1-800-QUIT-NOW booklet, 211 flyer). ST is mailed at 3 timepoints including baseline and following completion of the 6- and 12-month follow-up assessments.

#### MAPS

MAPS has all ST components along with 6 proactive telephone counseling sessions delivered over 12 months. The timing of the telephone counseling sessions is flexible and determined jointly by the participant and the counselor. Each call lasts approximately 30 minutes. Calls are scheduled based on participants’ needs in negotiation with the MAPS counselor. For example, a participant who is not yet ready to quit might schedule a second call to occur many months later or to occur sooner if there are specific barriers that the individual wishes to address (eg, stress, social support, and family problems). Similarly, participants struggling with maintaining abstinence may request several calls in a shorter period of time to get through the problematic period, whereas others prefer a less compressed counseling schedule and may need less frequent help.

The MAPS counselor for this study has completed 20 hours of MAPS training and is able to deliver MAPS in both English and Spanish. To monitor deviation or drift from the MAPS treatment manual, the calls are digitally recorded and encrypted. A random sample of 10% are reviewed and coded using a modified version of the Motivational Interviewing Treatment Integrity (MITI) manual to ensure adequate competence and adherence to the motivational interviewing components of MAPS. The MITI manual [31] has empirically validated reliability and validity and is used to code sessions and ensure treatment fidelity. The protocol stipulates that if a counselor’s performance falls below the stipulated performance criteria, there will be additional training. MITI results are reviewed regularly throughout the study during supervision, and the instrument works well to ensure that counselors are utilizing the general motivational interviewing spirit. In addition, the MITI has been modified slightly for the current project to include coding of discussions around social cognitive/problem solving strategies and transitions between motivational enhancement and problem solving. To monitor implementation, weekly monitoring reports are reviewed to track call completion and follow-up rates.

#### Nicotine Replacement Therapy

All participants in both treatment groups are provided a 12-week supply of nicotine patches and lozenges when ready to quit. Included with the NRT, all participants receive educational materials describing potential side effects, proper use of the patch, and an illustration demonstrating the proper placement of the patch on the body. The nicotine patch and lozenge regimens are based on each participant’s self-reported smoking rate. Participants who smoke >10 cigarettes/day receive 8 weeks of 21-mg patches, 2 weeks of 14-mg patches, 2 weeks of 7-mg patches, and 12 weeks of 2-mg lozenges. Those who smoke <10 cigarettes/day receive 8 weeks of 14-mg patches, 4 weeks of 7-mg patches, and 12 weeks of 2-mg lozenges.

### Measures

#### Baseline Assessment

Individuals who met eligibility criteria and provided informed consent completed the baseline assessment either over the phone with a research coordinator or via a secure electronic link sent via email or SMS text message. REDCap was used to administer all questionnaires over the telephone, in person, and via a weblink. REDCap is a secure, web-based application designed to support data capture and utilizes a computer-administered self-interview format. This system is designed to comply with all HIPAA regulations. The baseline assessment included questionnaires assessing sociodemographics, smoking history, and nicotine dependence [32], cancer status (ie, cervical cancer vs high-grade cervical dysplasia diagnosis, cancer stage at diagnosis, time since diagnosis, current cancer stage, and treatment status), fear of cancer recurrence [33], health literacy [34], subjective numeracy [35], subjective social status [36], financial strain [37], motivation to quit smoking [38], reasons for quitting [39], sense of control [40], self-efficacy [41], coping inventory [42], loneliness [43], perceived stress [44], positive and negative affect [45], psychological distress [46], smoking dependence motives [47,48], smoking withdrawal symptoms [49], quality of life [50], and health utilities/health-related quality of life [51,52].

#### Follow-up Assessments

Participants are asked to complete follow-up assessments by telephone or a secure emailed weblink at 3, 6, 12, and 18 months post baseline. All baseline measures are included in the follow-ups with the exception of sociodemographics and nicotine dependence. In addition, smoking status is assessed on the basis of recommendations from the Society for Research on Nicotine and Tobacco (SRNT), including both prolonged and point-prevalence abstinence [53]. Prolonged abstinence refers to abstinence beginning with the initiation of treatment and including a grace period. The prolonged abstinence measure utilizes the SRNT recommendation for determining relapse (ie, 7 consecutive days of smoking or smoking in each of 2 consecutive weeks). In addition, 2 point-prevalence abstinence measures are evaluated: (1) no smoking during the previous 7 days and (2) no smoking during the previous 30 days. The primary outcome is 7-day point prevalence abstinence from smoking at 18 months.

Participants who self-report 7-day point prevalence abstinence at any follow-up assessment are mailed a prepaid envelope with instructions for providing the saliva sample and a saliva collection kit. Research staff contact participants by phone to ensure the arrival of the packet, review the contents of the packet, and answer any questions participants may have about collecting a saliva specimen. Although cotinine cannot comprehensively validate the various abstinence definitions and timeframes, the most comprehensive review on biochemical validation concluded that misreporting is typically very low (~2%), and adjustment for misreporting almost never influences analyses regarding relative treatment efficacy [54]. As such, our biochemical validation procedures are well justified both scientifically and practically.

### Data Analysis Plan

#### Analysis Overview

The primary aim is to compare the efficacy of MAPS in facilitating smoking cessation with ST among women with a history of high-grade cervical dysplasia or cervical cancer. The primary outcome is 7-day point prevalence abstinence from smoking at the 18-month assessment. Logistic regression will be used with treatment (ST vs MAPS) as the predictor. The model will include as covariates those variables used in the minimization procedures (race/ethnicity, age, education, cigarettes per day, diagnosis at study enrollment, cervical cancer stage, treatment status, and time since diagnosis).

Secondary outcomes include 7-day point prevalence abstinence over time across the 3-, 6-, 12-, and 18-month assessments as well as 7-day point prevalence abstinence at 3, 6, and 12 months. Abstinence over time will be examined using generalized linear mixed model regression (GLMM) [55,56] with a logit link, and binomial variance function will be used to analyze the effects of MAPS. Treatment, month of assessment, and their interaction are the primary predictors with adjustment for relevant covariates. Similarly, analyses will be conducted to assess the aggregate effect of MAPS on the secondary outcomes of quit attempt and use of the quitline (both binary). For the continuous secondary outcomes of cigarettes per day and the purported mechanisms, linear mixed model analysis will be performed to evaluate treatment differences using the same predictors and covariates.

To manage missing data, multiple imputation under the Missing at Random assumption will be applied using a Markov Chain Monte Carlo method [57] via PROC MI in SAS (version 9.4, The SAS Institute) given the expected large numbers of nonmonotonic missing data patterns and auxiliary variables (eg, baseline measures that predict smoking status or missingness). In total, 20 data sets will be created. For smoking status, a post hoc adjustment [58] will be applied to implement an influence of Missing Not at Random (MNAR) (ie, *missing* is due to smoking). In recent publications [59], we have applied a small to medium effect size (Cohen *d*=0.35). This approach provides better parameter estimates and tests of hypotheses than does imputing missing equals smoking.

#### Sample Size Estimation

Our power analysis is based primarily on the comparison of 18-month abstinence rates between MAPS and ST using the full sample without attrition (N=300; n=150 per group). All power analyses assume a significance level of .05 and a 2-sided test. Based on the Treating Tobacco Use and Dependence Clinical Practice Guideline [60], we estimated that abstinence for ST would be approximately 10%. Using a chi-square test to examine the effect of treatment on abstinence at 18 months, a sample size of 300 (n=150 per group) will provide 80% power to detect an overall treatment effect that corresponds to an abstinence rate of 21.9% in MAPS. Analyses conducted using GLMM will have greater power to detect the same average differences over time.

#### Cost-effectiveness Analysis

The cost-effectiveness of MAPS will be carefully evaluated using state-of-the art methodologies. It should be noted that interventions targeted at expanding the population or increasing the intensity or duration of treatment often lead to an increase in health care utilization (and consequently costs); hence, an intervention may not be cost saving but still cost effective. Because health outcomes, costs, and efficient allocation of limited resources are paramount concerns, this study will yield crucial information necessary in determining whether MAPS should be widely implemented following the study.

Cost-effectiveness analyses (CEA) will compare the two interventions: ST and MAPS. The conventional CEA summarizes study findings in terms of the incremental cost-effectiveness ratio (ICER) [61,62]. The ICER, calculated as the difference in mean costs between the new and standard treatment divided by the difference in mean effectiveness between the new and standard treatment, estimates the additional resource consumption needed to achieve an increase in an additional unit of effectiveness. The ICER is then compared with a commonly cited or published threshold value associated with an intervention already found to be cost-effective to determine whether a new intervention is cost-effective. The net benefit approach, introduced more recently [63,64], transforms the ICER into the net benefit, defined as NB(λ) = λ • ΔE – ΔC, where λ represents a societal willingness-to-pay, ΔC represents the incremental costs, and ΔE represents the incremental effectiveness. We report the CEA results for both the conventional ICER and the cost-effectiveness acceptability curve [63,65]. The net benefit approach has been incorporated into a regression framework to allow for covariate adjustments and the examination of interaction effects in CEA [66]. This regression-based approach is relevant to our study because there may be moderating factors such as individual characteristics that affect the cost-effectiveness of interventions.

To facilitate comparing ICER estimated from our CEA with that from other published studies, we will include three effectiveness measures: number of quitters, years of life saved (YOLS), and quality-adjusted life year (QALY). The number of quitters in each treatment arm will be retrieved from the primary abstinence endpoint at month 18. We will extrapolate from abstinence to YOLS using a published algorithm that models YOLS per quitter for persons in various age-specific subgroups [67]. We will calculate QALY from the health utilities obtained from the EQ-5D [68].

We will compare the cost-effectiveness of the interventions in three time frames: short-term, mid-term, and long-term. The short-term and mid-term CEA will use “number of quitters” as the effectiveness measure and assess cost-effectiveness on the basis of information collected at months 3 and 6 (short-term), month 12 (mid-term), and month 18 (long-term), respectively. The long-term analysis will extrapolate the intervention effect to lifetime and use YOLS and QALY as the effectiveness measure. A 3% discount rate will be applied to costs and outcomes accrued in the second year and thereafter.

We will perform deterministic CEA on the basis of ICER and will apply the Bayesian approach to construct the cost-effectiveness acceptability curve and conduct probabilistic sensitivity analysis [69,70]. We will perform the Bayesian analysis using WinBUGS, with costs modeled as a gamma or lognormal distribution and abstinence from tobacco as a binomial distribution.

Finally, we will apply the regression-based CEA. Individual-level net benefit will be regressed on covariates, plus a binary variable indicating the ST versus MAPS arm. Using the ST arm as the reference group, the regression coefficient associated with the treatment binary variable will provide information on the cost-effectiveness of the MAPS intervention compared with ST.

## Results

This study was funded by the National Cancer Institute in 2015 and approved by the IRBs at the University of Texas MD Anderson Cancer Center, the University of Oklahoma Health Sciences Center, and Moffitt Cancer Center. Participant enrollment concluded at Moffitt Cancer Center in January 2020 (n=202), and follow-up data collection was completed in July 2021. Data analysis is ongoing.

## Discussion

There is a crucial need to provide cervical cancer survivors with evidence-based smoking cessation treatment designed to facilitate long-term cessation while addressing related survivorship issues. This need is enhanced by nationwide data, indicating that there are profound racial/ethnic and sociodemographic disparities in the incidence and mortality of cervical cancer [4-9]. For example, cervical cancer and cervical dysplasia survivors with lower socioeconomic status and limited social support are at an even greater risk for poor health outcomes. There is also widespread evidence suggesting that women who are members of racial/ethnic minority groups and with low socioeconomic status are known to disproportionately be faced with the health consequences of smoking [5,71,72]. Existing evidence suggests that these individuals may have greater difficulty quitting smoking [5,73-75]. Furthermore, disparities in tobacco use by socioeconomic status have increased over the last several decades despite widespread availability of free, effective cessation treatment. As such, women who are current smokers and who have a history of cervical cancer or high-grade cervical dysplasia represent a particularly vulnerable subgroup at substantially elevated risk.

This study represents the first large-scale smoking cessation treatment study designed to address the specific needs of high-grade cervical dysplasia and cervical cancer survivors using the MAPS approach. MAPS utilizes an innovative combination of motivational enhancement and cognitive-behavioral treatment techniques; is built around a structure derived from effective approaches to chronic care management and patient navigation; is designed for all individuals regardless of their readiness to change; and specifically targets cardinal mechanisms underlying tobacco use including motivation, agency/self-efficacy, and stress/negative affect. Furthermore, the creation of a wellness program for each individual makes MAPS particularly well suited for addressing the broader context of stressors and concerns faced by this vulnerable and underserved population [76].

Although previous research has supported the efficacy of MAPS for motivating quit attempts, increasing cessation, and preventing relapse [26-28], and motivationally based interventions have demonstrated efficacy for problematic alcohol use among individuals not ready to change [77,78], there are no empirically validated treatments that increase cessation among smokers who may not be ready to quit. It was anticipated that a fair proportion of women will not be ready to quit at the time of study enrollment [60], and, as such, the wellness program component of MAPS will enable these individuals to focus on other life issues (eg, stress, family issues, finances, adjustment to a cancer diagnosis, and fear of cancer recurrence). Thus, MAPS is designed to treat all individuals regardless of their readiness to change, thereby addressing this lack of evidence-based treatment.

In addition to being appropriate for individuals with different levels of motivation to change, MAPS has been designed to handle heterogeneity regarding time since cervical cancer diagnosis and treatment. For example, it is anticipated that women with more recent diagnosis and treatment experiences may be interested in discussing issues related to treatment side effects or fear of cancer recurrence, whereas women with more distal diagnoses and treatment experiences may prefer not to address such issues.

Prior to initiating the RCT, we conducted a series of in-depth interviews with cervical cancer survivors who currently smoked to gather feedback about how to best adapt MAPS for this specific population. Our results indicate that most participants attributed their diagnosis solely to human papillomavirus and did not believe that smoking had played a role in causing their cervical cancer. Participants suggested that the intervention include education about smoking and cancer and the benefits of quitting, help with planning for quitting, strategies for coping with cravings/withdrawal, social support, real-time support, a nonjudgmental and understanding counselor, tailoring, and follow-up. They recommended that negativity or judgment not be a part of the intervention. In-depth interview participants also indicated that it would be important to address stress, issues specific to cervical cancer survivorship, and lifestyle factors such as physical activity and healthy eating. Finally, they emphasized the importance of including NRT as part of the intervention [79].

This study has several unique strengths. First, this is the only RCT to target the specific needs of high-grade cervical dysplasia and cervical cancer survivors using the MAPS approach. Second, there are no empirically validated treatments that increase cessation among smokers who may not be ready to quit. Third, the delivery of MAPS via telephone offers a less resource-intensive modality while also minimizing participant burden.

In summary, this study will yield crucial information regarding the efficacy and cost-effectiveness of a MAPS approach for smoking cessation tailored to the specific needs of women with a history of high-grade cervical dysplasia or cervical cancer. Findings indicating that MAPS has substantially greater efficacy than existing evidence-based tobacco cessation treatments would have tremendous public health significance.

## Appendix

Multimedia Appendix 1 Peer review reports from the Psychosocial Risk and Disease Prevention Study Section - Center for Scientific Review (National Institutes of Health).

## Abbreviations

CEA: cost-effectiveness analysis
CIN: cervical intraepithelial neoplasia
GLMM: generalized linear mixed model regression
ICER: incremental cost-effectiveness ratio
IRB: institutional review board
MAPS: Motivation And Problem Solving
MI: motivational interviewing
MITI: Motivational Interviewing Treatment Integrity
MNAR: missing not at random
NRT: nicotine replacement therapy
QALY: quality-adjusted life years
RCT: randomized controlled trial
REDCap: Research Electronic Data Capture
SRNT: Society for Research on Nicotine and Tobacco
ST: standard treatment
YOLS: years of life saved
