# Supplementary material for: Evaluation of the Efficacy of a Smoking Cessation Intervention for Cervical Cancer Survivors and Women With High-Grade Cervical Dysplasia: Protocol for a Randomized Controlled Trial
Source: JMIR Res Protoc. 2021 Dec 30;10(12):e34502. doi: 10.2196/34502 (PMC8765796; doi:10.2196/34502)
Supplement: Multimedia Appendix 1 [file resprot_v10i12e34502_app1.pdf]

SUMMARY STATEMENT
(Privileged Communication)

PROGRAM CONTACT:
Elizabeth Ginexi
240-276-6765
lginexi@mail.nih.gov

Release Date: 09/26/2013

Application Number: 1 R01 CA172786-01A1

Principal Investigator

VIDRINE, JENNIFER IRVIN PHD

Applicant Organization: UT MD ANDERSON CANCER CTR

Review Group: PRDP
Psychosocial Risk and Disease Prevention Study Section

Meeting Date: 09/16/2013
Council: JAN 2014
Requested Start: 04/01/2014
RFA/PA: PA11-260
PCC: G7TC
Dual PCC: CM/RDX
Dual IC(s): DA

Project Title: Smoking Cessation for Cervical Cancer Survivors in a Safety Net Healthcare System
SRG Action: Impact Score: 20 Percentile: 8
Next Steps: Visit http://grants.nih.gov/grants/next_steps.htm
Human Subjects: 30-Human subjects involved - Certified, no SRG concerns
Animal Subjects: 10-No live vertebrate animals involved for competing appl.
Gender: 2A-Only women, scientifically acceptable
Minority: 1A-Minorities and non-minorities, scientifically acceptable
Children: 1A-Both Children and Adults, scientifically acceptable
Clinical Research - not NIH-defined Phase III Trial

Project Year	Direct Costs Requested	Estimated Total Cost
1	407,814	643,544
2	449,094	708,685
3	474,096	748,139
4	394,205	622,069
5	363,013	572,847
TOTAL	2,088,222	3,295,284

ADMINISTRATIVE BUDGET NOTE: The budget shown is the requested budget and has not been adjusted to reflect any recommendations made by reviewers. If an award is planned, the costs will be calculated by Institute grants management staff based on the recommendations outlined below in the COMMITTEE BUDGET RECOMMENDATIONS section.

1R01CA172786-01A1 Vidrine, JENNIFER

COMMITTEE BUDGET RECOMMENDATIONS

RESUME AND SUMMARY OF DISCUSSION: This application requests support to conduct a randomized two arm study to test a motivation and problem solving (MAP) intervention to promote smoking cessation in women with a history of cervical cancer. This work offers very high impact and high potential to inform new treatment strategies for both smoking cessation and cervical cancer prevention. This resubmission was extremely responsive to previous critiques. Reviewers noted many strengths: the outstanding and experienced research team and exceptional research environment; the significant and novel focus on smoking cessation, cervical cancer survivors and inclusion of an underserved minority target population; and an approach with a strong theoretical underpinning which includes a cost effectiveness analysis. A few weaknesses were discussed: missed opportunity to inform this work using previously collected pilot data; the decision to omit rather than revise the texting intervention is seen a weakness; concern about the lack of justification for the 18 month abstinence primary outcome time point; and the inclusion of nondaily smokers may complicate the study interpretation. Overall, this outstanding application that has some minor weaknesses that could readily be addressed.

DESCRIPTION (provided by applicant): The prevalence of smoking among cervical cancer survivors is strikingly high and smoking has been strongly linked to cervical cancer. Estimates of smoking prevalence among cervical cancer survivors have been reported in the literature as ranging from 44% to 48%. Continuing to smoke after a cancer diagnosis is associated with an increased risk of cancer recurrence, second primary cancers, and other smoking-related morbidities. This study will evaluate the efficacy and cost-effectiveness of a theoretically- and empirically- based "Motivation And Problem-Solving" (MAPS) approach for promoting and facilitating smoking cessation among cervical cancer survivors in a large, safety net public healthcare system. MAPS is a comprehensive, dynamic, and holistic intervention that incorporates empirically supported cognitive behavioral and social cognitive theory-based treatment strategies within an overarching motivational framework. MAPS was designed to be appropriate for all smokers regardless of their motivation to change, and views motivation as dynamically fluctuating from moment to moment throughout the behavior change process. Because MAPS comprehensively addresses multiple issues important to the individual and relevant to change through the creation of a Wellness Program (e.g., stressors, anxiety, depression, family conflicts, finances, fear of cancer recurrence), we believe it is particularly appropriate for treating this population. Participants will be current smokers who have been diagnosed with cervical cancer (N=300) recruited through the Harris Health System. Participants will be followed for a period of 18 months, and will be randomly assigned to one of two treatment groups: 1) Standard Treatment [ST] or 2) MAPS. ST will consist of a mailed packet of materials including a letter referring smokers to the Texas Quitline, free nicotine replacement therapy when they are ready to quit, and standard self-help materials. ST will be mailed a total of 3 times (at Baseline, 6 and 12 months). MAPS will consist of ST plus 6 proactive telephone counseling sessions delivered over a 12-month period. All assessments will be administered via telephone and occur at baseline and 3, 6, 12 and 18 months after baseline. The primary outcome is abstinence from tobacco at 18 months and the secondary outcomes are abstinence from tobacco at other assessments, as well as quit attempts, cigarettes per day, use of the Texas Quitline across all post-baseline assessments, and cost-effectiveness. The specific aims are to: 1) Compare the efficacy of a MAPS approach to promoting and facilitating smoking cessation to ST among cervical cancer survivors; 2) Assess the effects MAPS on hypothesized treatment mechanisms (motivation, agency, and stress/negative affect) and the role of those mechanisms in mediating MAPS effects on abstinence from smoking; and 3) Compare the cost-effectiveness of MAPS and ST.

PUBLIC HEALTH RELEVANCE: Smoking prevalence among cervical cancer survivors is strikingly high. Smoking has been strongly linked to cervical cancer and continuing to smoke after a cancer

diagnosis is associated with an increased risk of cancer recurrence, second primary cancers, and other smoking-related morbidities. This study will evaluate the efficacy and cost-effectiveness of a theoretically- and empirically-based "Motivation And Problem-Solving" (MAPS) approach to promoting and facilitating smoking cessation among cervical cancer survivors within a large, safety net public healthcare system.

CRITIQUE 1:

Significance: 3
Investigator(s): 1
Innovation: 1
Approach: 3
Environment: 1

Overall Impact: In this revised application, investigators propose to help cervical cancer survivors quit smoking. The investigators were responsive to many of the previous concerns. They simplified their study design to test one intervention against a control arm. They dropped the texting element and also decreased overlap among investigators, while adding a gynecological oncologist. They provided stronger justification for their prevalence estimates of smoking among cervical cancer survivors. They now include Spanish-speaking Latinas. They also make 18-month abstinence their primary outcome. This application has many strengths. It is the first to attempt to help cervical cancer survivors quit smoking. The investigators present a strong case that cervical cancer survivors have a high rate of smoking, which contributes to recurrence and other significant health problems. The investigators present a strong theoretical underpinning for their intervention. The investigative team is strong and can design and implement this study. The team has a solid plan for insuring that the MAPS intervention will be culturally adapted. There are some concerns with the application. The investigators still do not present pilot data of this intervention with cervical cancer survivors. The investigators state that cervical cancer survivors are interested in quitting, but this does not provide feasibility and acceptability data for the proposed intervention. Previous reviewers were concerned about the long term effects of the MAPS interventions given no studies have shown effects at 12 months. The investigators did not provide more data that the intervention will have the long term effects they desired. The investigators are including daily and non-daily smokers and justify their decision well. Given they are including non-daily smokers, they need to adjust their intervention. For future resubmitted applications, it would be helpful if the investigators made revisions clear by either putting a line at the side of the paragraph or putting new text in bold or italics. Overall, the investigators are addressing an important area.

1. Significance:

Strengths

- Investigators make a compelling case that interventions are needed to promote smoking cessation among cervical cancer survivors.
- The investigators include English and Spanish speaking survivors.

Weaknesses

- The investigators still do not present pilot data of their intervention among cervical cancer survivors. This diminishes the potential impact of the proposed project.

2. Investigator(s):

Strengths

- The investigators have much experience implementing smoking cessation interventions in various populations including cancer survivors.
- Drs. Vidrine and Wetter have published extensively together.
- The team has expertise in culturally adapting interventions for Latinos.

Weaknesses

- None noted

3. Innovation:

Strengths

- This will be the first to design a smoking cessation program for cervical cancer survivors.

Weaknesses

- None noted

4. Approach:

Strengths

- Investigators can feasibly recruit their sample.
- Investigators include both English and Spanish speakers, which increases the generalizability of their results.
- Investigators designate 18 months as their primary outcome, which allows for testing for maintenance of intervention effects.
- Investigators include prolonged abstinence along with 7 and 30-day point prevalence. These measures are important, particularly for non-daily smokers.
- The investigators propose a stream-lined study design.
- The investigators have a solid plan for culturally adapting their intervention.

Weaknesses

- Investigators have no pilot data for their intervention in this population.
- The investigators do not present data showing long term effects of other interventions that have used the MAPS framework. The data from the MOM trial showed effects at 6 months postpartum. The gold standard for postpartum return to smoking trials is 12 months postpartum.
- The investigators include non-daily smokers (in last submission, participants were required to smoke at least one daily cigarette). Non-daily smokers differ from daily smokers on key factors. The investigators do not discuss how their intervention will be tailored for non-daily smokers (e.g., smokers not identifying as smokers, ability of smoker to control when and where they smoke, lower physical dependence, etc.).
- Rather than randomizing based on cigarettes/day, it might make more sense to randomize based on daily vs. non-daily smoker. Investigators propose randomizing based on 9 variables, which seems like too many.
- The investigators will provide nicotine patches. Other forms of NRT might be more appropriate for light and non-daily smokers.

5. Environment:

Strengths

- Environments at MD Anderson will support this project.

Weaknesses

- None noted.

Protections for Human Subjects:

Acceptable Risks and/or Adequate Protections

Data and Safety Monitoring Plan (Applicable for Clinical Trials Only):

Acceptable

Inclusion of Women, Minorities and Children:

G2A - Only Women, Acceptable

M1A - Minority and Non-minority, Acceptable

C1A - Children and Adults, Acceptable

Vertebrate Animals:

Not Applicable (No Vertebrate Animals)

Resubmission:

- The investigators were responsive to many of the previous concerns. They changed their study design, specified one primary outcome, and added investigators to the team. They still do not present pilot data and now include non-daily smokers, which complicates the study.

Budget and Period of Support:

Recommend as Requested

CRITIQUE 2:

Significance: 1
Investigator(s): 1
Innovation: 2
Approach: 2
Environment: 1

Overall Impact: This study is a well written resubmission. Important, innovative and interesting, the project has likelihood for exerting a sustained and strong influence in the field and thus has a high impact factor. Although there are some weaknesses, these are minor. The general overall framework and approach are adequate. The population is significant, as cervical cancer survivors who smoke are a unique and important population. Cervical cancer is one of the leading cancers among Hispanic women. Smoking aggravates a disease that is preventable. There is an addition of an investigator with

cultural expertise, although the investigator is junior. Given the low numbers of intermittent smokers, and low numbers of smokers among Hispanic women, it is uncertain that the applicants will be able to obtain the numbers in the population within the given timeframe. However this is an experienced team, with a very strong environment, which is experienced in tobacco control and cancer, and has the capacity to carry this application forward successfully. Given the high potential for impact in this study, and the implications for potentially changing the field and critically advancing our knowledge on these issues, the proposal stands to yield high impact.

1. Significance:

Strengths

- This study focuses on individuals who continue to smoke after a cervical cancer diagnosis who are particularly at high risk of cancer recurrence, second primary cancers, and other smoking-related morbidities.
- Addresses an important problem among high risk patients
- A large number of cervical cancer survivors are current smokers
- Cervical cancer affects younger women with a median age of 49 years
- Addressed some serious ethnic disparities

Weaknesses

- None noted.

2. Investigator(s):

Strengths

- Very strong team of investigators
- Dr. Vidrine has a solid record of tobacco research including addressing issues of literacy and smoking risk and nicotine dependence
- The research team has strong expertise and experience in research methods.
- Excellent tobacco researchers
- Excellent expertise in cancer control
- Strong statistical team

Weaknesses

- None noted.

3. Innovation:

Strengths

- MAPS provides a framework for behavior change that Integrates treatment elements from both motivational interviewing (MI) and social cognitive theory.
- MAPS is structured around a Wellness Program that addresses a wide array of concerns and barriers to change, similar to patient navigation programs

Weaknesses

- Not particularly innovative, although population is innovative
- Had originally a text-messaging component. Previous review had concerns about the texting, and is disappointing to see the texting removed. Would have preferred to see the investigators addressing why it was justified and simplified it and explained it better in terms of feasibility and recruitment. Much of the innovation was based on the texting they dropped.

4. Approach:

Strengths

- Uses motivational interviewing and social cognitive theory
- Addresses a wide range of concerns
- Structured around a 3-wellness program.
- Issues of retention are still not well addressed over the one year period
- Concerns regarding missing data are well addressed
- Shorter measures and less lengthy intervention assessments reduce potential subject fatigue concerns
- Over half of the women being recruited are either Hispanic Latino or Blacks, populations at high risk for cervical cancer
- Has potential for being cost effective intervention
- Well-developed program over the last 15 years
- Evidence of its success over usual care and proven effective in 3 RCT
- Mixed methods approach which includes a series of focus groups including in Spanish
- A strength of this work is its sensitivity to needs of cervical cancer survivors

Weaknesses

- Not clear regarding outreach of various minority groups, and need place special attention to instrumentation in the intervention in order to effectively reach Hispanic and Black women to recruit the sample. For example, the application lacks detail in how motivational interviews will be tailored to particular Hispanic or Black audiences – all seems the same except that one focus group will be conducted in Spanish.
- Concerns about the duration of the intervention still provides concerns over retention
- Concerns over recruiting smokers who feel they are not addicted – low and non-daily intermittent smokers, so different from daily smokers. Application needs greater attention to detail in this regard and understanding these two populations more profoundly. However, because this is an experienced team of tobacco researchers, this is a weakness that they can easily resolve.
- Concern about sample recruitment: Since the sample will include Hispanic populations there are concerns over the number of women in that population who both smoke and have cervical cancer. Even though cervical cancer is prevalent the rates of smoking are low, thus will there be enough of a universe to obtain the numbers needed in the population for the study?
- Did not see measurement for acculturation which might be an important variable given the Hispanic sample and that will have at least one focus group in Spanish. The inclusion of cultural, ethnic and racial minorities is not clearly spelled out, nor has been elaborated upon in

detail in the proposal. Special attention to these populations is critical, in recruitment and especially for retention over an extended period of time.

5. Environment:

Strengths

- University of Texas, MD Anderson Cancer Center has the necessary equipment, space, and facilities to support this study
- MD Anderson is a strong and capable institution
- Outstanding and exceptional environment

Weaknesses

- None noted.

Protections for Human Subjects:

Acceptable Risks and/or Adequate Protections

- Risks and protections are adequate in the sample of 300 subjects

Data and Safety Monitoring Plan (Applicable for Clinical Trials Only):

Acceptable

- This work has an elaborate but effective data monitoring plan

Inclusion of Women, Minorities and Children:

G2A - Only Women, Acceptable

M1A - Minority and Non-minority, Acceptable

C1A - Children and Adults, Acceptable

- Study among females is adequate given focus on cervical cancer doing.

Vertebrate Animals:

Not Applicable (No Vertebrate Animals)

Biohazards:

Not Applicable (No Biohazards)

Resubmission:

- The applicants have been responsive to prior reviewer concerns

Resource Sharing Plans:

Acceptable

- A good plan attached

Budget and Period of Support:

Recommend as Requested

Additional Comments to Applicant (Optional):

- Given Dr. Vidrine's experience in mobile technology, she might consider a future application where that component is addressed. May consider a minority supplement or other way to measure the role of texting (now dropped in this resubmission) as this was one of the most innovative and exciting aspects of this study. It is highly recommended that Dr. Vidrine consider a future application that includes this, or include as an offshoot of this application.

CRITIQUE 3:

Significance: 2
Investigator(s): 3
Innovation: 1
Approach: 3
Environment: 1

Overall Impact: This application proposes a randomized controlled trial of a motivation and problem solving (MAPS) intervention to promote smoking cessation among cervical cancer survivors. The project addresses an understudied and clinically important issue. The investigative team is excellent, as is the environment at MD Anderson Cancer Center. The actual intervention is not particularly innovative, but this is a minor concern in view of the novel population of focus. The significance of the project is slightly reduced by the lack of consideration of how the proposed intervention might be incorporated into standard clinical care. The proposed approach is very sound and the outcomes, measures, and analytic plan are all very good. There are still a few concerns regarding the proposed approach, although they are relatively minor in the context of an otherwise very strong application.

1. Significance:

Strengths

- High rates of continued smoking among cervical cancer survivors are of public health concern.
- There is a need for efficacious smoking cessation interventions targeting cervical cancer survivors.
- The proposed telephone-based intervention approach facilitates potential dissemination of the intervention

Weaknesses

- Little consideration is given to how the proposed intervention might be integrated into broader systematic approaches to promote the delivery of smoking cessation advice and quit assistance by health care providers.

2. Investigator(s):

Strengths

- Dr. Vidrine is well-qualified to lead the proposed randomized controlled trial

- The research team included investigators with complementary areas of expertise, including health economics, biostatistics, gynecologic oncology, and smoking cessation

Weaknesses

- Insufficient justification is provided for the need to support both a biostatistician and a statistical analyst throughout the entire period of the project. This is a minor issue.

3. Innovation:

Strengths

- Smoking cessation interventions targeting cervical cancer survivors are lacking.

Weaknesses

- The actual intervention that will be used has been employed in prior research, but overall this is a minor issue.

4. Approach:

Strengths

- The MAPS intervention is suitable for individuals with varying levels of motivation for quitting smoking.
- Good consideration of treatment fidelity issues.
- Focus on English- and Spanish-speaking individuals
- Efforts to ensure that the intervention is culturally relevant to Hispanic individuals.
- Biochemical verification of reported smoking abstinence using salivary cotinine
- Good randomization plan
- Sound plans for examining the cost-effectiveness of the intervention
- Robust statistical plan for handling missing data

Weaknesses

- The number of in-depth interviews and focus groups to be conducted may not be sufficient to obtain adequate feedback regarding the proposed intervention.
- One-quarter of the study participants will be African American. While efforts to ensure the cultural relevance of the intervention to Hispanic individuals are outlined, it is not clear that similar efforts will be made with regard to African Americans.
- While the study will be powered (based on N = 300) to detect a treatment effect corresponding to a 10 percent abstinence rate at 18 months in the standard treatment group and 21.9 percent in the MAPS group, it is not clear what the actual expected abstinence rate is for the MAPS group. What level of smoking abstinence would be required to determine the intervention successful?
- Insufficient justification is provided for focusing on abstinence at the 18 month time point as the primary outcome. This is the first study of the proposed intervention for cervical cancer survivors and the 18 month time point is 6 months after treatment completion. Thus, it may be more prudent to utilize an earlier time point as the primary outcome and to consider the 18 month time point in a more exploratory manner.

- It would be helpful to have additional information about the standard self-help materials that will be distributed to all participants.

5. Environment:

Strengths

- The resources and environment at MD Anderson Cancer Center are excellent and very well-suited to the proposed project.

Weaknesses

- None noted.

Protections for Human Subjects:

Acceptable Risks and/or Adequate Protections

- The potential study-related risks and protections in place are outlined appropriately.

Data and Safety Monitoring Plan (Applicable for Clinical Trials Only):

Acceptable

Inclusion of Women, Minorities and Children:

G2A - Only Women, Acceptable

M1A - Minority and Non-minority, Acceptable

C1A - Children and Adults, Acceptable

- No men will be recruited because the study focuses on survivors of cervical cancer. Children aged 18-20 years will be recruited, which is appropriate. The minority recruitment rate is acceptable.

Vertebrate Animals:

Not Applicable (No Vertebrate Animals)

Biohazards:

Not Applicable (No Biohazards)

Resubmission:

- The investigators were very responsive to the previous set of reviews. The numerous changes include the new 2-arm design (instead of the prior 4-arm design), inclusion of Spanish-speaking participants, revised accrual estimates, and addition of a gynecologic oncologist. A lingering concern is that the primary outcome is smoking abstinence assessed at the 18 month outcome.

Budget and Period of Support:

Recommended budget modifications or possible overlap identified:

- The requested biostatistical support for the project is high.

THE FOLLOWING RESUME SECTIONS WERE PREPARED BY THE SCIENTIFIC REVIEW OFFICER TO SUMMARIZE THE OUTCOME OF DISCUSSIONS OF THE REVIEW COMMITTEE ON THE FOLLOWING ISSUES:

PROTECTION OF HUMAN SUBJECTS (Resume): ACCEPTABLE. There are no concerns.

INCLUSION OF WOMEN PLAN (Resume): ACCEPTABLE. As a study of cervical cancer survivors, a study sample of females only is justified.

INCLUSION OF MINORITIES PLAN (Resume): ACCEPTABLE. Black, Hispanic and White race and ethnic groups will be enrolled in this study.

INCLUSION OF CHILDREN PLAN (Resume): ACCEPTABLE. Children 18 to 20 and adults will be eligible for this study.

COMMITTEE BUDGET RECOMMENDATIONS: Reviewers noted that the requested biostatistical support for the project is excessive.

NIH has modified its policy regarding the receipt of resubmissions (amended applications). See Guide Notice NOT-OD-10-080 at http://grants.nih.gov/grants/guide/notice-files/NOT-OD-10-080.html.
The impact/priority score is calculated after discussion of an application by averaging the overall scores (1-9) given by all voting reviewers on the committee and multiplying by 10. The criterion scores are submitted prior to the meeting by the individual reviewers assigned to an application, and are not discussed specifically at the review meeting or calculated into the overall impact score. Some applications also receive a percentile ranking. For details on the review process, see http://grants.nih.gov/grants/peer_review_process.htm#scoring.

MEETING ROSTER

Psychosocial Risk and Disease Prevention Study Section
Risk, Prevention and Health Behavior Integrated Review Group
CENTER FOR SCIENTIFIC REVIEW
PRDP
September 16, 2013 - September 17, 2013

CHAIRPERSON

EPSTEIN, LEONARD H, PHD
SUNY DISTINGUISHED PROFESSOR
DEPARTMENT OF PEDIATRICS
DIVISION OF BEHAVIORAL MEDICINE
STATE UNIVERSITY OF NEW YORK AT BUFFALO
BUFFALO, NY 14214

MEMBERS

ABRANTES, ANA M, PHD
PROFESSOR
DEPARTMENT OF PSYCHIATRY AND
HUMAN BEHAVIOR, ALPERT MEDICAL
SCHOOL, BUTLER HOSPITAL
BROWN UNIVERSITY
PROVIDENCE, RI 02906

AYALA, GUADALUPE X, PHD
PROFESSOR
INSTITUTE FOR BEHAVIORAL AND
COMMUNITY HEALTH
SAN DIEGO STATE UNIVERSITY
SAN DIEGO, CA 92123

BAEZCONDE-GARBANATI, LOURDES ALBERTINA, MPH,
PHD
ASSOCIATE PROFESSOR
DEPARTMENT OF PREVENTIVE MEDICINE
AND SOCIOLOGY, KECK SCHOOL OF MEDICINE
NORRIS COMPREHENSIVE CANCER CENTER
UNIVERSITY OF SOUTHERN CALIFORNIA
LOS ANGELES, CA 90033

BASKIN, MONICA L, PHD
ASSOCIATE PROFESSOR
DIVISION OF PREVENTIVE MEDICINE
UNIVERSITY OF ALABAMA AT BIRMINGHAM
BIRMINGHAM, AL 35294

BOUTELLE, KERRI N, PHD
PROFESSOR
DEPARTMENT OF PEDIATRICS
AND PSYCHIATRY
UNIVERSITY OF CALIFORNIA, SAN DIEGO
LA JOLLA, CA 92037

COUPS, ELLIOT J, PHD
ASSOCIATE PROFESSOR
DEPARTMENT OF HEALTH EDUCATION
RUTGERS, THE STATE UNIVERSITY OF NEW JERSEY
ROBERT WOOD JOHNSON MEDICAL SCHOOL
NEW BRUNSWICK, NJ 08901

DAVISON, KIRSTEN , PHD
ASSOCIATE PROFESSOR
DEPARTMENT OF NUTRITION
HARVARD SCHOOL OF PUBLIC HEALTH
BOSTON, MA 02115

DODD, VIRGINIA JONES, PHD *
ASSOCIATE PROFESSOR
DEPARTMENT OF COMMUNITY DENTISTRY
AND BEHAVIORAL SCIENCES
UNIVERSITY OF FLORIDA
GAINESVILLE, FL 32611

FERRY, ROBERT , MD *
PROFESSOR AND CHIEF
DIVISION OF PEDIATRIC
DEPARTMENT OF ENDOCRINOLOGY AND METABOLISM
LE BONHEUR CHILDRENS HOSPITAL
UNIVERSITY OF TENNESSEE HEALTH SCIENCES
CENTER
MEMPHIS, TN 38103

FOCHT, BRIAN CARL, PHD *
ASSOCIATE PROFESSOR
DEPARTMENT OF KINESIDOLOGY
COMPREHENSIVE CANCER CENTER
AND SOLOVE RESEARCH INSTITUTE
THE OHIO STATE UNIVERSITY
COLUMBUS, OH 43210

LEAHEY, TRICIA M, PHD
ASSISTANT PROFESSOR
WEIGHT CONTROL AND DIABETES RESEARCH CENTER
MIRIAM HOSPITAL
BROWN MEDICAL SCHOOL
PROVIDENCE, RI 02903

LEVINE, MICHELE D, PHD
ASSOCIATE PROFESSOR
DEPARTMENT OF PSYCHIATRY
UNIVERSITY OF PITTSBURGH
PITTSBURGH, PA 15213

MADSEN, KRISTINE A, MD *
ASSISTANT PROFESSOR
SCHOOL OF PUBLIC HEALTH, JOINT MEDICAL PROGRAM
AND PUBLIC HEALTH NUTRITION
UNIVERSITY OF CALIFORNIA BERKELEY
BERKELEY , CA 94720

MORLAND, KIMBERLY B, PHD
ASSOCIATE PROFESSOR
DEPARTMENT OF PREVENTIVE MEDICINE
MOUNT SINAI SCHOOL OF MEDICINE
NEW YORK, NY 10029

PAGOTO, SHERRY L, PHD *
ASSISTANT PROFESSOR
DIVISION OF PREVENTIVE AND BEHAVIORAL MEDICINE
DEPARTMENT OF MEDICINE
UNIVERSITY OF MASSACHUSETTS MEDICAL SCHOOL
WORCESTER, MA 01655

PAUL, IAN M, MD *
PROFESSOR
DEPARTMENT OF PEDIATRICS
PENNSYLVANIA STATE COLLEGE OF MEDICINE
HERSHEY, PA 17033-085

POLLAK, KATHRYN I, PHD
ASSOCIATE PROFESSOR
DUKE CANCER PREVENTION
DETECTION AND CONTROL RESEARCH PROGRAM
DUKE UNIVERSITY MEDICAL CENTER
DURHAM, NC 27705

QUARELLS, RAKALE COLLINS, PHD
RESEARCH ASSOCIATE PROFESSOR
SOCIAL EPIDEMIOLOGY RESEARCH CENTER
MOREHOUSE SCHOOL OF MEDICINE
ATLANTA, GA 30310

SMITH, MICHAEL T, PHD
PROFESSOR
DEPARTMENT OF PSYCHIATRY AND BEHAVIORAL SLEEP
CENTER FOR BEHAVIOR AND HEALTH
SCHOOL OF MEDICINE
JOHNS HOPKINS UNIVERSITY
BALTIMORE, MD 21224

SPRING, BONNIE , PHD *
PROFESSOR
DEPARTMENT OF PREVENTIVE MEDICINE
NORTHWESTERN UNIVERSITY
CHICAGO, IL 60611

TATE, DEBORAH F, PHD
ASSOCIATE PROFESSOR
DEPARTMENT OF HEALTH BEHAVIOR
AND NUTRITION
UNIVERSITY OF NORTH CAROLINA
CHAPEL HILL, NC 27599

THOMPSON, DEBORAH I, PHD
ASSOCIATE PROFESSOR
USDA/ARS SCIENTIST/NUTRITIONIST
CHILDREN'S NUTRITION RESEARCH CENTER
BAYLOR COLLEGE OF MEDICINE
HOUSTON, TX 77030

WILFLEY, DENISE ELLA, PHD
PROFESSOR
DEPARTMENT OF PSYCHIATRY, MEDICINE
PEDIATRICS, AND PSYCHOLOGY
SCHOOL OF MEDICINE IN ST. LOUIS
WASHINGTON UNIVERSITY
ST. LOUIS, MO 63110

YAROCH, AMY L, PHD
EXECUTIVE DIRECTOR
GRETCHEN SWANSON
CENTER FOR NUTRITION
OMAHA, NE 68105

MAIL REVIEWER(S)

STICE, ERIC M, PHD
SENIOR RESEARCH SCIENTIST
OREGON RESEARCH INSTITUTE
EUGENE, OR 97403

SCIENTIFIC REVIEW ADMINISTRATOR

FITZSIMMONS, STACEY , PHD
SCIENTIFIC REVIEW OFFICER
CENTER FOR SCIENTIFIC REVIEW
NATIONAL INSTITUTES OF HEALTH
BETHESDA, MD 20892

GRANTS TECHNICAL ASSISTANT

NJOKU, PHILIP C
CENTER FOR SCIENTIFIC REVIEW
NATIONAL INSTITUTES OF HEALTH
BETHESDA, MD 20892

* Temporary Member. For grant applications, temporary members may participate in the entire meeting or may review only selected applications as needed.

Consultants are required to absent themselves from the room during the review of any application if their presence would constitute or appear to constitute a conflict of interest.
